# Malnutrition trends in Rohingya children aged 6–59 months residing in informal settlements in Cox’s Bazar District, Bangladesh: An analysis of cross-sectional, population-representative surveys

**DOI:** 10.1371/journal.pmed.1003060

**Published:** 2020-03-31

**Authors:** Eva Leidman, Md. Lalan Miah, Alexa Humphreys, Leonie Toroitich-van Mil, Caroline Wilkinson, Mary Chelang'at Koech, Henry Sebuliba, Muhammad Abu Bakr Siddique, Oleg Bilukha

**Affiliations:** 1 Division of Global Health Protection, Center for Global Health, Centers for Disease Control and Prevention, Atlanta, Georgia, United States of America; 2 Action Against Hunger Bangladesh, Dhaka, Bangladesh; 3 United Nations High Commissioner for Refugees, Geneva, Switzerland; 4 United Nations High Commissioner for Refugees, UNHCR Sub-Office (BGDCO), Cox's Bazar, Bangladesh; 5 United Nations Children’s Fund, Dhaka, Bangladesh; International Organization for Migration, SRI LANKA

## Abstract

**Background:**

More than 700,000 ethnic Rohingya have crossed the border from Rakhine State, Myanmar to Cox’s Bazar District, Bangladesh, following escalated violence by Myanmar security forces. The majority of these displaced Rohingya settled in informal sites on previously forested land, in areas without basic infrastructure or access to services.

**Methods and findings:**

Three cross-sectional population-representative cluster surveys were conducted, including all informal settlements of Rohingya refugees in the Ukhia and Teknaf Upazilas of Cox’s Bazar District. The first survey was conducted during the acute phase of the humanitarian response (October–November 2017), and the second and third surveys were conducted 6 (April–May 2018) and 12 (October–November 2018) months later. Anthropometric indices (weight, height, mid-upper arm circumference [MUAC], oedema) and haemoglobin (Hb) were measured in children aged 6–59 months following standard procedures. Final samples for survey rounds 1, 2, and 3 (R1, R2, and R3) included 1,113, 628, and 683 children, respectively, of which approximately half were male (50.7%–53.5% per round) and a third were 6–23 months of age (32.4%–33.3% per round). Prevalence of global acute malnutrition (GAM) as assessed by weight for height in R2 (12.1%, 95% CI: 9.6–15.1) and R3 (11.0%, 95% CI: 8.4–14.2) represent a significant decline from the observed prevalence in R1 (19.4%, 95% CI: 16.8–22.3) (*p* < 0.001 for both comparisons). Overall, the prevalence of anaemia significantly declined (*p* < 0.001) between the first 2 rounds (47.9%, 95% CI: 44.1–51.7 and 32.3%, 95% CI: 27.8–37.1, respectively); prevalence increased significantly (*p* = 0.04) to 39.8% (95% CI, 34.1–45.4) during R3 but remained below R1 levels. Reported receipt of both fortified blended foods (12.8%) and micronutrient powders (10.3%) were low during R1 but increased significantly (*p* < 0.001 for both) within the first 6 months to 49.8% and 29.9%, respectively. Although findings demonstrate improvement in anthropometric indicators during a period in which nutrition programme coverage increased, causation cannot be determined from the cross-sectional design.

**Conclusions:**

These data document significant improvements in both acute and micronutrient malnutrition among Rohingya children in makeshift settlements. These declines coincide with a scaleup of services aimed at prevention and treatment of malnutrition. Ongoing activities to improve access to nutritional services may facilitate further reductions in malnutrition levels to sustained below-crisis levels.

## Abstract 2

Eva Leidman and colleagues reveal the extent of malnutrition in Rohingya children at the time of displacement.

## Author summary

**Why was this study done?:**

- Surveys were organised in the months following the mass displacement of Rohingya from Rakhine State, Myanmar to Cox’s Bazar District, Bangladesh to collect information on health and nutrition necessary to inform the ongoing humanitarian response.
- Evidence regarding the effects of large-scale, multisectoral humanitarian responses in the acute phase of an emergency on nutritional status of children remains limited.

**What did the researchers do and find?:**

- The study presents data from 3 population-representative surveys: the first conducted 2 months (October–November 2017) following mass displacement, and the other two 6 (April–May 2018) and 12 (October–November 2018) months later. These surveys included 1,113, 628, and 683 children, respectively.
- Prevalence of wasting among children was 19.4% in the first survey but declined significantly during the subsequent 6 months. Nearly half of children assessed in the first survey were anaemic (47.9%). Prevalence of anaemia was also significantly lower in the second survey but rebounded slightly by survey round 3.
- Coverage of major humanitarian assistance programmes—including receipt of household food rations, fortified blended foods, and micronutrient powders—also increased significantly.

**What do these findings mean?:**

- The data provide an indication that, as of October–November 2017, the nutritional status of Rohingya children in makeshift settlements of Cox's Bazar District, Bangladesh, exceeded the World Health Organization (WHO) global emergency thresholds for both wasting and anaemia. Nutritional status improved in the period 6–12 months after initial assessment.
- Improvements in nutritional status corresponded with a scaleup of humanitarian interventions, including those to prevent and treat cases of malnutrition.

## Introduction

More than 700,000 Rohingya have fled to Bangladesh since August 25, 2017, following an escalation of violence and ethnic persecution in Rakhine State, Myanmar [1]. Because this influx by far overwhelmed the capacity of the 2 pre-existing refugee camps located near the Bangladesh–Myanmar border (Kutupalong and Nayapara), the majority of Rohingya have settled in makeshift settlements in Cox’s Bazar District, Bangladesh. These makeshift settlements cover an area of approximately 12.8 million square meters (m2), with an average density of 11 m2 of usable land per person, more than 3 times the maximum density of refugee settlements recommended by the United Nations High Commissioner for Refugees (UNHCR) [2]. Built on previously forested land interspersed with rice paddies, these informal settlements lacked basic infrastructure such as access to roads, water points, and health facilities [3].

Whilst scaling up efforts to provide shelter and services, humanitarian partners raised concerns about the health and nutritional status of Rohingya children, given evidence of poor nutritional status among children in Rakhine State compounded by the stress of the multiday journey to reach Bangladesh. Data from the 2015–16 Myanmar Demographic Health Survey reported that Rakhine State had the worst nutritional status of children in Myanmar: 38% of children less than 5 years of age were chronically malnourished, and 14% were acutely malnourished [4]. Data from Kutupalong Refugee Camp following the August displacement provided evidence that 24% of children in the camp were acutely malnourished, above the World Health Organization (WHO) emergency level of 15%, suggesting a further deterioration of nutritional status with displacement or upon arrival in Bangladesh [5,6].

An initial nutrition assessment of the makeshift settlements was organised in October–November 2017, during the acute phase of the response. At the time, 4 inpatient and 31 independent outpatient centres were admitting children with acute malnutrition for treatment in these informal settlements, each serving catchment populations of approximately 20,000 people [7]. Rohingya households arrived with few possessions and remained largely dependent on emergency rations consisting of rice, vegetable oil, and lentils [8]. Infectious diseases, including measles and diphtheria, threatened to aggravate the situation.

During the following 12 months, an additional 2 inpatient and 27 outpatient centres for treatment of acute malnutrition were opened in the makeshift settlements, the majority of which started providing services during the first half of 2018 [9]. During the same time period, 29 targeted and blanket supplemental feeding programmes providing nutritional support for children 6–59 months of age were also opened [9]. Emergency rations were gradually replaced with e-vouchers to allow for purchase of food, including fresh vegetables, from designated markets [8]. Nutrition partners prioritised enhancing house-to-house screening of children for acute malnutrition, preventive distributions of micronutrient-fortified foods to young children, and counselling on breastfeeding and complementary feeding for caregivers. Concurrently, additional primary health centres, latrines, and water points, as well as other physical infrastructure, were constructed. Ten vaccination campaigns were organised between September 2017 and May 2018, including measles–rubella for individuals 6 months to 15 years (2 rounds), bivalent oral polio for children 5 years and younger (2 rounds), oral cholera for all individuals over 1 year (3 rounds), and diphtheria–tetanus with bivalent oral polio for those aged 1.5 months to 15 years (3 rounds) [10]. By 2018, nearly $990 million US dollars had been provided by international donors to fund the response to the Rohingya refugee crisis [11].

The primary aim of this study was to estimate prevalence of acute malnutrition and anaemia in Rohingya children aged 6–59 months shortly after the August 25, 2017 mass displacement and again approximately 6 and 12 months later, following a scaleup of health and nutritional services as well as other assistance programmes in the makeshift settlements. Such a temporal comparison allows for a description of the severity of the nutritional situation during the initial phase of the response, as well as its evolution corresponding with increased availability of humanitarian interventions that followed. This study is based on data from representative cross-sectional surveys conducted by Action Against Hunger (AAH), together with the Bangladesh Government, United Nations Children's Fund (UNICEF), UNHCR, the World Food Programme (WFP), Save the Children, and the US Centers for Disease Control and Prevention (CDC), designed to collect evidence to inform ongoing humanitarian programmes. Each assessment was planned as a one-off event responding to information needs of the humanitarian response. The presented comparison and trend analysis were planned following completion of the third round of data collection. No data-driven changes to analysis took place.

## Methods

**Fig 1:**
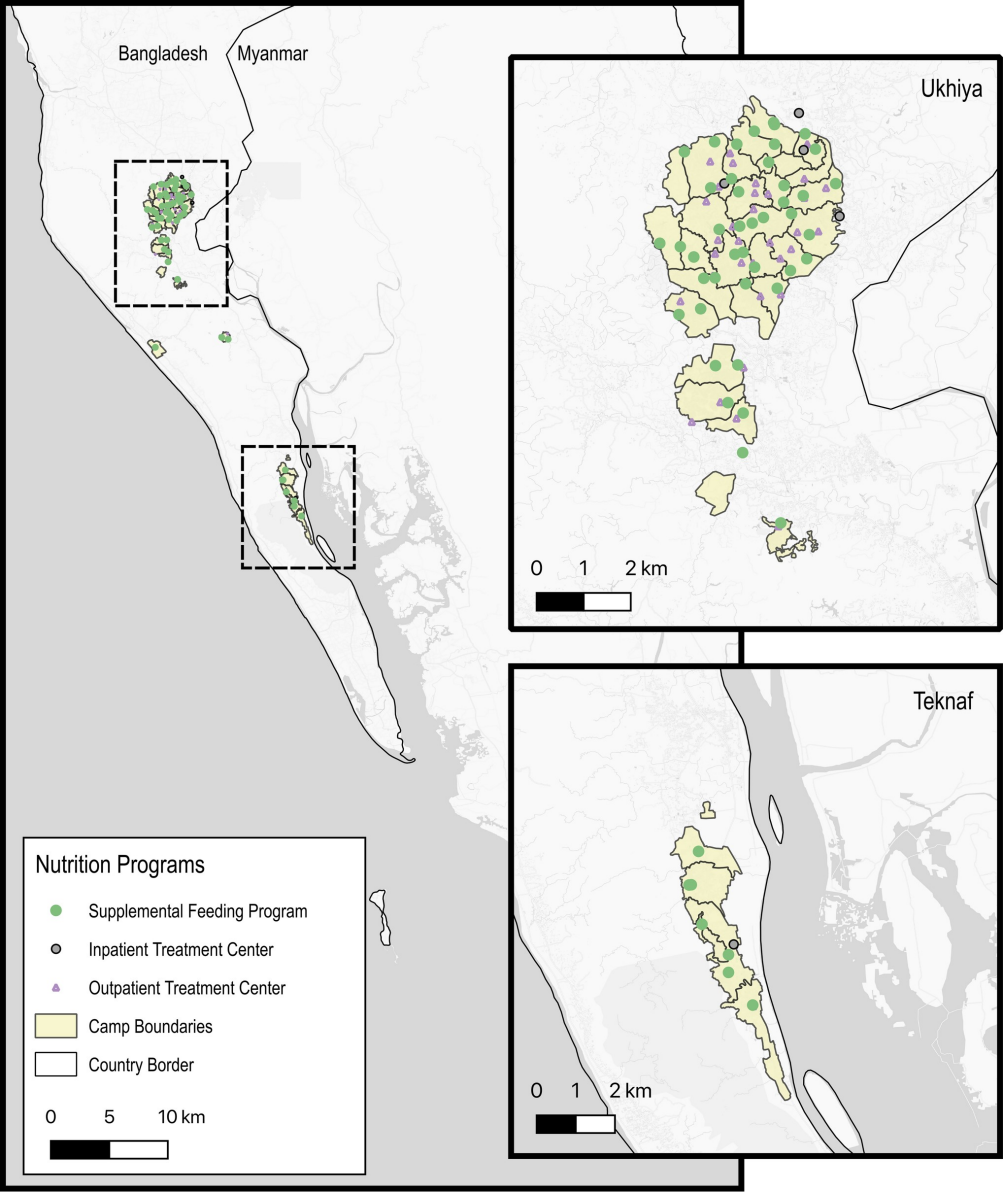
Map of nutrition centres in the makeshift settlements. Location of nutrition programmes in makeshift settlements of Cox’s Bazar, Bangladesh as of August 2018. Base maps including refugee camp boundaries obtained from the Humanitarian Data Exchange.

Three cross-sectional two-stage cluster surveys assessing Rohingya children aged 6–59 months were conducted in informal settlements in the Ukhia and Teknaf Upazilas of Cox’s Bazar District (Fig 1). Data were collected during October 29–November 20, 2017 for the first survey (R1), during April 28–May 9, 2018 for the second survey (R2), and during October 20–November 8, 2018 for the third survey (R3). The selection of samples for each round was independent of other rounds. Population estimates for makeshift and spontaneous settlements were obtained from the Intersectoral Coordination Group (ISCG) for R1 and from the International Organization for Migration (IOM) Needs and Population Monitoring database for R2 and R3. The estimated total population of these settlements at the time of the surveys was approximately 720,900, 904,700, and 867,700 during R1, R2, and R3, respectively [12,13].

A target sample size of 833 children selected from 1,344 households was calculated to achieve a precision of ±3.25% for global acute malnutrition (GAM) in R1. For R2 and R3, a target sample size of 463 children selected from 715 households and 505 children selected from 742 households were calculated, respectively. Sample size parameters are presented in S1 Table. Sample size parameters were updated for each round to reflect changing demographics, nutritional status, and programme needs. A larger sample was drawn during R1 to allow for disaggregation between Rohingya arriving before and after August 25, 2017, given meaningful differences in eligibility for humanitarian assistance. However, initial data suggesting no meaningful difference in nutritional status by time of arrival prompted a change in programme eligibility, and the distinction was determined not to be programmatically relevant for R2 and R3 [5]. Therefore, sample sizes for R2 and R3 were smaller because as the objective of disaggregating the results by time of arrival (before or after August 25, 2017) was no longer relevant. For all 3 surveys, clusters were selected using probability proportional to size. Within each cluster, households were mapped and enumerated prior to the survey to allow for random selection of households. All children aged 6–59 months residing in selected households were eligible for inclusion regardless of registration status or date of arrival in Bangladesh. Households were defined as a group of people living together and sharing resources, without regard for registration status, marriage, or biological relationship.

Anthropometric indices (weight, height, mid-upper arm circumference [MUAC], oedema) were measured following standard procedures [14]. Z-scores were calculated using 2006 WHO growth standards, and extreme outliers were excluded following standard WHO exclusion criteria [15]. Two definitions of GAM and severe acute malnutrition (SAM) were investigated, one based on weight-for-height z-score (WHZ) and another based on MUAC. GAM was defined as WHZ less than –2 or MUAC less than 125 mm and/or oedema. SAM was defined as WHZ less than –3 or MUAC less than 115 mm and/or oedema. Haemoglobin (Hb) was measured using HemoCue Hb 301 (HemoCue, Ӓngelholm, Sweden) following standard procedures. Children aged 6–59 months were classified as having severe or moderate anaemia (Hb < 10.0 g/dL) or mild anaemia (10 g/dl ≤ Hb < 11 g/dl) according to WHO thresholds [16]. Total anaemia (Hb < 11.0 g/dl) included cases of severe, moderate, and mild anaemia. Inclusion of additional indicators was informed by programmatic need.

The 2-week prevalence of 3 conditions were ascertained by caregiver self-report during the study. Diarrhoea was defined as the passage of 3 or more loose or liquid stools within a 24-hour period during the 2 weeks preceding the interview. Acute respiratory infection (ARI) was defined as cough with rapid or difficulty breathing and fever during the 2 weeks preceding the interview. The case definition for the third condition, fever without cough during the 2 weeks preceding the interview, changed between R1 and R2 to additionally exclude cases with rash during R2 and R3 to distinguish between cases of measles given an ongoing measles outbreak.

Rohingya households received either monthly rations or electronic vouchers. Rations contained 30 kg rice, 9 kg pulses (lentils) and 3 litres of fortified vegetable oil. The number of rations received each month was proportional to household size—households composed of 1–3 members received 1, those with 4–7 members received 2, etc. Vouchers were provided as biometric debit cards that could be used at shops operated by private-sector merchants contracted by the WFP selling a minimum of 18 mandatory food commodities, including fresh spinach, pumpkin, and dried fish. Receipt of household rations was assessed in all 3 rounds by reviewing ration cards and/or vouchers, but the means of distribution (by electronic voucher or otherwise) was only assessed in R2 and R3 because vouchers were not yet introduced at the time of the first survey. Breastfeeding practices and minimum dietary diversity were reported by the primary caregiver of each child; these indicators were assessed only in R1 and R2 because an in-depth infant and young child feeding assessment was separately planned in winter 2018.

Micronutrient powders (locally marketed as Pushtikona) were distributed as 1-g sachets house to house by humanitarian partners [17]. Fortified blended food distributed in the camps was Super Cereal Plus—Wheat Soya Blend (WSB++) (Michiels Fabrieken, Zulte, Belgium); children were eligible to receive 200 g/day, distributed biweekly or monthly. Receipt of micronutrient powders and enrolment in therapeutic programmes were assessed in all 3 rounds. Receipt of fortified blended foods were assessed only in R1 and R2. Receipt of fortified blended foods was verified by reviewing blanket and/or targeted supplemental feeding programme enrolment cards. Receipt of micronutrient powders, distributed quarterly, was assessed by caregiver recall following a visual prompt, using a recall period beginning in August 2017 for R1, January 2018 for R2, and June 2018 for R3. Enrolment in an outpatient therapeutic feeding programme (OTP) was confirmed using enrolment cards. Severely malnourished children were eligible for enrolment in therapeutic programmes; eligibility for all other programmes was by age rather than nutritional status. Minimum dietary diversity was assessed based on primary caregiver report using a standard WHO questionnaire and defined as consumption of at least 4 of 7 food groups on the day preceding the survey [18]. Questionnaires for all 3 rounds are publicly available [7,9,19].

Six teams of 4 members each (an interviewer, 2 anthropometry measurers, and a haemoglobin measurer) received 6 days of training prior to R1, including a standardisation test and a field test. Prior to R2 and R3, these teams received a 5-day refresher training. Team members all spoke Bangla and the local dialect (Chittagonian), which is very similar to the unwritten, oral dialect spoken by the Rohingya. Teams were accompanied by an interagency team of supervisors. Verbal informed consent was obtained before performing interviews and measurements.

WHZs were calculated using Emergency Nutrition Assessment (ENA) software [20]. Statistical analysis was performed using STATA (version 11.2) [21]. Estimates are adjusted for the sampling design. Pearson chi-squared tests were used to compare prevalence estimates from different survey rounds. A chi-squared statistic for trend (regression) is calculated to evaluate trend across the 3 rounds. Statistical significance was evaluated with two-sided tests with a significance threshold of 0.05. This study is reported as per the Strengthening the Reporting of Observational Studies in Epidemiology (STROBE) guideline (S1 STROBE Checklist). The Institutional Review Board of CDC determined this study to be nonresearch. Personal identifiers were not included in the final data set used for analysis.

## Results

A total of 1,343 households were randomly selected in R1, of which 38 (2.8%) were absent and none refused. During R2, a total of 715 households were randomly selected, of which 39 (5.5%) were absent and one (0.1%) refused. During R3, of the 742 planned households, 53 (7.1%) were absent and 25 refused (3.4%). Mean household size increased from 4.7 in R1 to 5.0 in R2 to 5.4 in R3. There were no meaningful differences with respect to other demographic characteristics of households assessed (Table 1). Children under 5 represented approximately a fifth of all household members in all 3 surveys. Among households surveyed, approximately one in five had no adult male over 18 years of age. The majority of households reported arriving in Bangladesh since August 25, 2017 (Table 1).

**Table 1:** Demographic profile of sampled households and children—Bangladesh, 2017–2018.

	October–November 2017 (R1)	April–May 2018 (R2)	October–November 2018 (R3)
**Demographics of selected households**			
Household size, mean (SD)	4.71 (2.09)	5.04 (2.30)	5.38 (2.38)
% of children under 5, (n/N) %	(1,249/6,146) 20.3%	(687/3,404) 20.2%	(741/3,573) 20.7%
% of female-headed households, (n/N) %^a^	(242/1,305) 18.54%	(127/675) 18.81%	(113/664) 17.02%
**Date of arrival of selected households, (n/N) %**			
Prior to August 25, 2017	(193/1,305) 14.79%	(44/675) 6.52%	(62/664) 9.34%
August 25, 2017 to date of interview	(1,112/1,305) 85.21%	(631/675) 93.48%^b^	(602/664) 90.66%^b^
**Demographics of children 6–59 months, (n/N) %**			
*Age*			
6–23 months	(361/1,113) 32.43%	(207/628) 32.96%	(227/682) 33.28%
24–59 months	(752/1,113) 67.57%	(421/628) 67.04%	(455/682) 66.72%
*Sex*			
Male	(589/1,113) 52.92%	(336/628) 53.50%	(336/682) 50.73%
Female	(524/1,113) 47.08%	(292/628) 46.50%	(346/682) 49.27%

**Abbreviations:** R1, round 1; R2, round 2; R3, round 3; SD, standard deviation.

^a^Female-headed households defined as households without a male aged 18 years or older.

^b^For both R2 and R3, only 1 surveyed household arrived after December 31, 2017.

The final sample included 1,113 children aged 6–59 months in R1, 628 children in R2, and 683 children in R3. Of these 1,113 eligible children residing in consenting households during R1, 1,087 (97.7%) were measured, and 1,110 (99.7%) of caregivers consented to interviews on behalf of eligible children. In R2, 600 (95.5%) of 628 eligible children were measured, and 100% of caregivers consented to interviews. In R3, 640 (93.8%) of 683 eligible children were measured, and caregivers for all but one child consented to interviews. No extreme outliers for anthropometric indicators were excluded in R1 and R2. In R3, WHZ values for 2 children for whom height could not be assessed were excluded.

Prevalence of GAM as assessed by WHZ declined significantly between R1 and R2, from 19.4% during R1 to 12.1% during R2 (*p* < 0.001). The subsequent decline in R3 to 11.0% was not significantly different from R2 (*p* = 0.584). The prevalence of SAM also decreased each round; however, the differences between each round were not significant. No cases of oedema were identified during any of the surveys. Similarly, the prevalence of GAM as assessed by MUAC declined significantly, from 8.6% during R1 to 4.3% during R2 (*p* < 0.001) and subsequently to 3.1% during R3 (*p* = 0.224 for comparison with R2), whereas the declines per round in SAM as assessed by MUAC were only significant between R2 and R3 (Table 2).

**Table 2:** Prevalence of GAM and anaemia among Rohingya children 6–59 months of age in makeshift and informal settlements—Bangladesh, 2017–2018.

	October–November 2017 (R1)			April–May 2018 (R2)			October–November 2018 (R3)			*p*-Value Trend
Indicator	n/N	Mean^a^	% (95% CI)	n/N	Mean^a^	% (95% CI)	n/N	Mean^a^	% (95% CI)	
***GAM***										
**Total (WHZ < –2)**										
Children aged 6–59 months	211/1,087	–1.20	19.4% (16.8–22.3)	72/596^b^	–1.01	12.1% (9.6–15.1)1	70/638	–0.95	11.0% (8.4–14.2)1	<0.001
Children aged 6–23 months	105/351	–1.45	29.9% (24.7–35.8)	39/196	–1.26	19.9% (14.6–26.6)^1^	34/216	–1.08	15.7% (11.2–21.7)^1^	0.001
Children aged 24–59 months	106/736	–1.08	14.4% (11.8–17.5)	33/400	–0.89	8.3% (6.3–10.8)1	36/442	–0.90	8.5% (5.9–12.2)^1^	0.004
**Severe (WHZ < –3)**										
Children aged 6–59 months	34/1,087	–	3.1% (2.3–4.3)^1^	13/596	–	2.2% (1.2–3.9)^1,2^	7/638	–	1.1% (0.4–2.8)2	0.007
**Total (MUAC < 125 mm)**										
Children aged 6–59 months	93/1,087	137.5	8.6% (6.8–10.7)	26/600	142.2	4.3% (3.2–5.8)1	20/640	145.1	3.1% (1.9–5.0)^1^	<0.001
Children aged 6–23 months	78/350	131.6	22.3% (17.4–28.0)	22/197	136.2	11.2% (8.0–15.3)1	19/217	137.5	8.8% (5.3–14.2)^1^	<0.001
Children aged 24–59 months	15/737	143.6	2.0% (1.2–3.4)^1^	4/403	147.5	1.0% (0.4–2.5)^1^	1/423	148.99	0.2% (0.0–1.7)^1^	0.008
**Severe (MUAC < 115 mm)**										
Children aged 6–59 months	14/1,087	–	1.3% (0.8–2.1)^1^	3/600	–	0.5% (0.2–1.5)^1^	0/640	–	0%	0.002
***Anaemia***										
**Total anaemia (Hb < 11.0 g/dl)**										
Children aged 6–59 months	518/1,082^c^	10.97	47.9% (44.1–51.7)	193/598^d^	11.46	32.3% (27.8–37.1)	253/636	11.14	39.8% (34.1–45.4)	<0.001
Children aged 6–23 months	215/349	10.53	61.6% (55.8–67.1)^1^	102/196	10.84	52.0% (44.0–60.0)^1^	115/216	10.71	53.2% (44.7–61.7)^1^	0.034
Children aged 24–59 months	303/733	11.17	41.3% (37.5–45.3)	91/402	11.76	22.6% (17.9–28.2)	138/420	11.36	32.9% (26.6–39.1)	0.002
**Severe or moderate anaemia (Hb < 10 g/dl)**										
Children aged 6–59 months	185/1082	–	17.1% (14.6–19.9)^1^	76/598	–	12.7% (10.0–16.0)	116/636	–	18.2% (14.1–23.2)^1^	0.809

**Abbreviations:** CI, confidence interval; GAM, global acute malnutrition; HAZ, height-for-age z-score; Hb, haemoglobin; MUAC, mid-upper arm circumference; R1, round 1; R2, round 2; R3, round 3; WHZ, weight-for-height z-score.

For pairwise tests comparing rounds, values in the same row sharing the same superscript number are not significantly different from each other (*p* < 0.05).

^a^Mean of the underlying continuous variable (WHZ, MUAC, HAZ, and Hb, respectively).

^b^Four children who were present and consented to measurement had physical disabilities that precluded measurement of height.

^c^Five children who were present and consented to anthropometric measurement did not provide consent for measurement of capillary blood for Hb assessment.

^d^Two children who were present and consented to anthropometric measurement did not provide consent for measurement of capillary blood for Hb assessment.

In all 3 surveys, prevalence of GAM as assessed by MUAC was less than half the prevalence as assessed by WHZ. Among all children identified as having GAM by either WHZ or MUAC, 60.8% of children were identified only by WHZ during R1, 68.3% during R2, and 61.5% in R3. Similarly, among all children identified as having SAM by either criterion, 62.2% in R1, 78.6% in R2, and all children in R3 were identified only by WHZ.

Total anaemia among children aged 6–59 months declined significantly between R1 (47.9%) and R2 (32.3%) (*p* < 0.001) but increased significantly (*p* = 0.04) to 39.8% during R3. Mean haemoglobin value increased from 10.97 to 11.46 and then declined to 11.14 g/dL. Changes were more pronounced among children 24–59 months than among children 6–23 months. Prevalence of moderate and severe anaemia followed the same trends (Table 2). Four cases of severe anaemia were observed: two in R1, one in R2, and one in R3.

Two-week prevalence of diarrhoea declined significantly (*p* < 0.001) from 41.3% to 20.9% between R1 and R2 before increasing significantly (*p* = 0.007) to 28.4% in R3, whereas two-week prevalence of ARI progressively declined between rounds from 57.7% in R1 to 26.1% in R2 to 10.9% in R3 (*p* < 0.001 for all pairwise comparisons) (Table 3). Two-week prevalence of fever without cough was 25.1% in R1. Two-week prevalence of fever without cough or rash was 40.0% in R2 and 38.0% in R3 (*p* = 0.58).

**Table 3:** Two-week prevalence of caregiver-reported morbidity among Rohingya children 6–59 months of age in makeshift and informal settlements—Bangladesh, 2017–2018.

	October–November 2017 (R1)		April–May 2018 (R2)		October–November 2018 (R3)	
Indicator	n/N	% (95% CI)	n/N	% (95% CI)	n/N	% (95% CI)
Diarrhoea	458/1,110	41.3 (36.5–46.2)	131/628	20.9 (17.4–24.8)	194/682	28.4 (24.5–32.4)
ARI with fever	640/1,110	57.7 (52.7–62.4)	164/628	26.1 (21.1–31.9)	73/682	10.9 (7.1–14.6)
Fever without cough (or rash)^a^	280/1,110	25.5 (20.5–30.6)	251/628	40.0 (34.6–45.6)^b^	259/682	38.0 (33.0–43.0)^b^

**Abbreviations:** ARI, acute respiratory infection; CI, confidence interval; R1, round 1; R2, round 2; R3, round 3.

^a^Case definition in R2 and R3 (only) excludes cases with rash.

^b^Values indicated with a superscript are not significantly different from each other; all other pairwise comparisons between rounds were significant (*p* < 0.05).

Indicators of infant and young child feeding practices were only assessed in R1 and R2; they remained largely unchanged between rounds (Table 4). Continued breastfeeding at one year was nearly universal (97.3% in both R1 and R2). Continued breastfeeding at 2 years was also high—71.1% in R1 and 62.5% in R2. However, only half of children under 6 months were exclusively breastfed during the day preceding the survey (56.1% and 50.0% in R1 and R2), a nonsignificant difference between rounds (*p* = 0.50). Consumption of a diet achieving minimum dietary diversity among children 6–23 months of age remained low—8.3% in R1 and 12.6% in R2.

The study documented significant changes in access to food and nutritional services targeted to all households or all age-eligible children (Table 5). The proportion of caregivers who reported that their children were receiving fortified blended foods as part of blanket or therapeutic feeding programme increased significantly from 12.8% in R1 to 49.8% in R2 (*p* < 0.001). Similarly, the proportion of children 6–59 months who reported receipt of micronutrient powders increased from 10.3% to 29.9% (*p* < 0.001) between R1 and R2 and to 58.7% in R3 (*p* < 0.001). Receipt of general household food rations increased from 82.2% to 98.1% (*p* < 0.001) and remained high (94.9%) in R3. During the months between data collection periods, electronic vouchers were gradually being phased in as an alternative to food distributions. During R2 and R3, an estimated 17.8% and 18.5% of households were receiving rations in the form of vouchers. The proportion of eligible children enrolled in OTPs increased over time, with significant increases in R3 relative to R1 (*p* = 0.008) and R2 (*p* = 0.024); however, estimates are likely unstable given small samples.

**Table 4:** Breastfeeding practices and dietary diversity among Rohingya children 6–23 months of age in makeshift and informal settlements—Bangladesh, 2017–2018.

**Indicator**	**October–November 2017 (R1)**		**April–May 2018 (R2)**		***p*-Value**
*Breastfeeding practices*	n/N	% (95% CI)	n/N	% (95% CI)	
Exclusive breastfeeding, children 0–5 months	74/132	56.1% (45.2–66.4)	29/58	50.0% (34.3–65.7)	0.50
Continued breastfeeding at 1 year, children 12–15 months	71/73	97.3% (89.2–99.4)	36/37	97.3% (83.6–99.6)	0.99
Continued breastfeeding at 2 years, children 20–23 months	32/45	71.1% (54.7–83.4)	25/40	62.5% (44.6–77.6)	0.44
***Dietary diversity*, *children 6–23 months***					
Minimum dietary diversity (4 of 7 food groups)^a^	30/361	8.3% (5.2–13.0)	26/207	12.6% (3.4–18.4)	0.17

**Abbreviations:** CI, confidence interval; R1, round 1; R2, round 2.

^a^Consumption of 4 of 7 food groups—1) grains, roots, or tubers; 2) legumes or nuts; 3) dairy products; 4) fresh foods; 5) eggs; 6) vitamin-A–rich fruits and vegetables; and 7) other fruits and vegetables—during the day preceding the survey.

**Table 5:** Receipt of nutritional services among children 6–59 months of age and households in makeshift and informal settlements—Bangladesh, 2017–2018.

	October–November 2017 (R1)		April–May 2018 (R2)		October–November 2018 (R3)		*p*-Value Trend
Indicator	n/N	% (95% CI)	n/N	% (95% CI)	n/N	% (95% CI)	
**Receipt of nutritional services, children aged 6–59 months**							
Receipt of fortified blended foods	142/1,110	12.8% (8.7–18.4)	313/628	49.8% (40.0–59.7)	–	–	<0.001
Received micronutrient powders^a^	114/1,110	10.3% (7.1–14.6)	188/628	29.9% (22.2–39.0)	400/682	58.7% (49.1–68.2)	<0.001
Eligible children enrolled in OTP^b^	6/38	15.8% (6.9–32.2)^1^	3/14	21.4% (6.4–52.1)^1^	2/6	33.3% (2.7–90.1)^1^	0.3038
**Receipt of nutritional services, households**							
Receiving household food rations^c^	1,066/1,297^d^	82.2% (75.1–87.6)	662/675	98.1% (96.1–99.1)^1^	630/664	94.9% (89.8–100)^1^	<0.001
Receiving household food rations in the form of electronic vouchers	–	–	120/675	17.8% (10.3–29.0)^1^	123/664	18.5% (8.7–28.3)^1^	0.723

**Abbreviations:** CI, confidence interval; OTP, outpatient therapeutic feeding programme; R1, round 1; R2, round 2; R3, round 3

For pairwise tests comparing rounds, values in the same row sharing the same superscript number are not significantly different from each other (*p* < 0.05).

^a^Recall period between August 25, 2017 and date of the interview for R1. Recall period of January 1, 2018 to date of the interview for R2. Recall period between June 15, 2018 to date of the interview for R3.

^b^Children aged 6–59 months with MUAC <115 mm, WHZ < –3, and/or bilateral pitting oedema were eligible for enrolment in an OTP.

^c^October–November 2017 assessment based on self-reported receipt of a household ration including (at a minimum) rice. April–May 2018 and October–November 2018 based on observation of a general food distribution ration card and/or an electronic voucher.

^d^Eight households registered as refugees with the UNHCR excluded from the analysis given differences in eligibility for household food rations.

## Discussion

Malnutrition among the sample of Rohingya children in makeshift settlements of Cox's Bazar District, Bangladesh exceeded global emergency thresholds in October–November 2017, shortly after mass displacement from Rakhine State, Myanmar. The prevalence of GAM exceeded the WHO emergency threshold of 15%, and the prevalence of anaemia exceeded the 40% threshold of a severe public health problem [6,18]. Wasting improved significantly by April–May 2018 and remained below emergency levels 1 year following the acute phase of the emergency. The same trend in wasting was observed when measured either by low WHZ or low MUAC. Because data collection in both R1 and R3 occurred in October–November, declines observed are unlikely to be due only to seasonal improvements in food availability [22].

Whilst improved substantially compared to initial levels, prevalence of wasting remains ‘high’ by WHO global standards. Further improvements require sustained efforts identifying children for treatment. In all 3 survey samples, over 60% of GAM and SAM cases were identified by WHZ only. Discordance in diagnosis of acute malnutrition by WHZ and MUAC among children from Bangladesh, Myanmar, and elsewhere in the world has been previously documented [5,23]. Since 2005, global guidelines have endorsed the use of WHZ and MUAC as independent criteria for admission into selective nutrition feeding programmes [24]. However, national protocols in many countries, including Bangladesh, recommend admission on only one criterion (MUAC). The evidence that most acutely malnourished Rohingya children are identified only by WHZ has prompted a review and revision of nutrition treatment protocols used at nutrition centres in the camps to include both MUAC and WHZ criteria [25].

The prevalence of anaemia declined significantly between R1 and R2. However, in contrast to wasting, the prevalence of anaemia subsequently increased in R3. Round to round variation in prevalence of anaemia was only significant among older children (24–49 months); prevalence declined almost by half (from 41.3% to 22.6%) between R1 and R2 and rebounded to 32.9% in R3. Changes in prevalence among younger children (6–23 months) were not significant, and the prevalence remained virtually unchanged between R2 and R3. Children in the older age category may be more responsive to changes in both food availability as well as access to humanitarian assistance, potentially benefitting more from the scaleup of micronutrient supplementation programmes (fortified blended food and micronutrient powder distributions), as well as suffering more from limited availability of food in the post-monsoon season (October–November). Poorer dietary diversity in October–November has been reported previously [26]. Whilst there are many markets of various size in and around the camp with regular supply of staple items, availability and cost of vegetables is seasonal, with less than half of the markets reportedly selling fresh vegetables in November 2017 [27]. Neither breastfeeding practices nor dietary diversity among children 6–23 months changed significantly between first and second survey rounds. The finding that the prevalence of anaemia among these younger children remained consistently over 50% in all rounds suggests a need for further efforts targeting young child feeding.

Trends in wasting, anaemia, and morbidity indicators provide evidence of the severity of the crisis in its initial stage and changes in health and nutritional status during the subsequent 6 and 12 months as humanitarian programmes scaled up to meet the identified needs. Coverage of nutritional programmes was measured to monitor this scaleup of humanitarian interventions and inform ongoing activities. Whilst the interventions likely contributed to the changes in biological indicators observed, in the context of a large-scale humanitarian response, in which a number of multisectoral interventions are delivered in parallel, changes in morbidity cannot be directly attributed to any particular cause.

Three ‘blanket’ interventions targeting either all children aged 6–59 months or all households were assessed: receipt of fortified blended foods, receipt of micronutrient powders, and receipt of general household rations. Increases in coverage of these interventions were all highly significant. However, half of age-eligible children did not receive fortified blended foods in R2, and more than a third did not receive micronutrient powders in R3, suggesting room for improvement. Active outreach in makeshift settlements to educate caregivers on the availability and benefits of preventive services, as well as minimising distance between households and distribution points, may help to further improve programme coverage. Coverage of electronic vouchers for food rations was heavily concentrated in certain clusters, suggesting a need to expand access to areas of the camps not previously targeted. Additionally, surveys assessed the proportion of children with SAM enrolled in therapeutic programmes. Changes in coverage of these programmes lagged that of the blanket intervention programmes. Many treatment centres were constructed immediately preceding R2. Efforts to ensure utilisation of treatment programmes require active community outreach, including identification of eligible children through house-to-house screening, which takes longer to scale up [28].

Improvements in nutritional status also corresponded with reductions in two-week prevalence of diarrhoea and ARI with fever, both of which were highest in R1. Trends in diarrhoea are generally consistent with that from the Early Warning and Response System (EWARS) facility-based surveillance, which reported a general decline in diarrhoea between the end of December 2017 and the middle of August 2018, at which point cases incidence increased, likely corresponding with the monsoon rains [29]. The first heavy rains in 2018 were in late April, with the peak rains in July and August [30]. Throughout the study period, the prevalence of diarrhoea greatly exceeded the national estimate of Bangladeshi children from 2018 [31]. Of interest, the dramatic and highly significant decline in ARIs observed in our study was not observed at health facilities reporting in EWARS, potentially because of differences in reference age group (all ages in EWARS reports), as well as care-seeking patterns [32]. Trends in fever without cough should be interpreted with caution given the change in case definition between R1 and R2, as well as potential changes in the sensitivity of self-reported fever following a confirmed measles outbreak.

This study is subject to several limitations. First, measures of Hb are inadequate to differentiate between iron deficiency and other forms of anaemia; high prevalence of thalassemia (β-thalassemia or Hb E), as well as deficiencies in other micronutrients (e.g., zinc, vitamin A), have previously been documented in Bangladesh [26,33]. Generalisability of findings on anaemia aetiology among Bangaladeshi to Rohingya children is unknown. Estimation of deficiencies in other micronutrients was beyond the scope of these emergency surveys. Second, our study represents nutritional status at the time of the assessments. However, the situation in the camps remains fluid. There remains a need to monitor the situation, given ongoing displacement, the possibility of changes in nutritional status in response to seasonal variation in market availability of different foods, outbreaks of infectious diseases, and changes in access to humanitarian services. Third, the protocol to verify enrolment in OTPs against admission cards may result in an underestimation of coverage. Finally, causality of association between the scaleup of humanitarian interventions and the improvements in wasting and anaemia cannot be determined from cross-sectional surveys. The presented data document improvements in nutritional status that coincided with improvements in access to blanket preventive nutrition programmes (household rations, fortified blended foods, and micronutrient powders) and therapeutic feeding programmes, as well as other public health interventions not assessed, including those directed at improving access to safe water, sanitation, and shelter. These multisectoral efforts to improve food security, ensure access to uncontaminated drinking water, control outbreaks, provide uninterrupted access to quality healthcare, improve community cohesion, and improve shelter quality in the camps have also likely contributed to reducing malnutrition.

### Conclusions

The presented data were collected just 2 months following one of the largest forcible displacement of the past decade and in regular intervals during the subsequent year, providing unique information rarely available in the context of a humanitarian crisis and the subsequent response. The findings provide an indication that the nutritional status of Rohingya children in makeshift settlements in Cox's Bazar District, Bangladesh was critical shortly following displacement but improved in the period 6–12 months after initial assessment. These improvements in nutritional status coincided with an increased coverage of household food rations, distributions of fortified blended foods and micronutrient powders, and other public health interventions. However, despite these documented successes, the increase in prevalence of anaemia 1 year following displacement, and the fact that the prevalence of wasting remains notably higher than that of the Bangladeshi population in the Chittagong Division underscore the need for ongoing monitoring [31]. The presented data suggest that sustained efforts are needed to ensure all eligible children are enrolled in available treatment and preventive services.

## Supporting information

S1 STROBE Checklist STROBE statement—Checklist of items that should be included in reports of cross-sectional studies. STROBE, Strengthening the Reporting of Observational Studies in Epidemiology
(DOCX)

S1 Table Sample size parameters for surveys of Rohingya children in makeshift and informal settlements—Bangladesh, 2017–2018. (DOCX)

## Abbreviations

AAH: Action Against Hunger
ARI: acute respiratory infection
CDC: Centers for Disease Control and Prevention
ENA: Emergency Nutrition Assessment
EWARS: Early Warning and Response System
GAM: global acute malnutrition
Hb: haemoglobin
IOM: International Organization for Migration
ISCG: Intersectoral Coordination Group
MUAC: mid-upper arm circumference
OTP: outpatient therapeutic feeding programme
SAM: severe acute malnutrition
STROBE: Strengthening the Reporting of Observational Studies in Epidemiology
UNHCR: United Nations High Commissioner for Refugees
UNICEF: United Nations Children’s Fund
WFP: World Food Programme
WHO: World Health Organization
WHZ: weight-for-height z-score
