# Supplementary material for: Malnutrition trends in Rohingya children aged 6–59 months residing in informal settlements in Cox’s Bazar District, Bangladesh: An analysis of cross-sectional, population-representative surveys
Source: PLoS Med. 2020 Mar 31;17(3):e1003060. doi: 10.1371/journal.pmed.1003060 (PMC7108721; doi:10.1371/journal.pmed.1003060)
Supplement: S1 Table — (DOCX) [file pmed.1003060.s002.docx]

**S1 Table. Sample size parameters for surveys of Rohingya children in makeshift and informal settlements— Bangladesh, 2017-2018**

| **Parameters** | Oct -Nov 2017 (R1) |  | April-May 2018 (R2) |  | Oct -Nov 2018 (R3) |
| --- | --- | --- | --- | --- | --- |
| Estimated Prevalence of GAM (%) | 22% |  | 20% |  | 12% |
| Desired precision | ±3.25 |  | ±4.5 |  | ±3.5 |
| Design Effect | 1.3 |  | 1.4 |  | 1.4 |
| Estimated proportion of children aged 6-59 months per household | 0.74 |  | 0.73 |  | 0.81 |
| Expected non-response | 10% |  | 10% |  | 6% |
| Minimum sample size calculated | 1,335 households |  | 706 households |  | 738 households |
| Sample size planned | 1,344 households  (96 clusters x 14 households) |  | 715 households  (55 clusters x 13 households) |  | 742 households  (53 clusters x 14 households) |

Using the above parameters, sample size was calculated as:

N = Z_α/2_^2^ ­*p*(1-p) / d^2^ * DEFF

Where:

Z_α/2_^2^ is the critical value of the Normal distribution at α/2 (e.g. for a confidence level of 95%, α is 0.05 and the critical value is 1.96)

d is the relative desired precision / margin of error

p is the sample proportion

DEFF is the design effect
